# Dr. Clair S. Parkhill

**Published:** 1909-09

**Authors:** 


Dr. Clair S. Parkhill, of Hornell, one of the best known and skilful physicians of the Southern Tier, died suddenly in his carriage, July 20, 1909, aged 64 years. Dr. Parkhill had been attending a patient on Bennett Street and when entering his carriage told the driver to hurry home as he did not feel well. Before his home was reached he expired. Dr. Parkhill was prominent in many medical associations and had held executive offices in the leading medical associations of the state. He was a member of the staff of St. James's and Mercy Hospitals, and of Steuben Sanitarium, and was surgeon for the Erie Railway at Hornell. He also had held several prominent city offices, having been mayor in 1884. He is survived by his wife and one daughter, Mrs. Blake B. Babcock.

